# Intraperitoneal insulin administration in pigs: effect on circulating insulin and glucose levels

**DOI:** 10.1136/bmjdrc-2020-001929

**Published:** 2021-01-15

**Authors:** Ilze Dirnena-Fusini, Marte Kierulf Åm, Anders Lyngvi Fougner, Sven Magnus Carlsen, Sverre Christian Christiansen

**Affiliations:** 1 Department Clinical and Molecular Medicine, Faculty of Medicine and Health Sciences, Norwegian University of Science and Technology, Trondheim, Norway; 2 Department of Endocrinology, St Olavs Hospital Universitetssykehuset i Trondheim, Trondheim, Norway; 3 Department of Engineering Cybernetics, Faculty of Information Technology and Electrical Engineering, Norwegian University of Science and Technology, Trondheim, Norway

**Keywords:** insulin, animal experimentation, blood glucose, diabetes mellitus, type 1

## Abstract

**Introduction:**

The effect of intraperitoneal insulin infusion has limited evidence in the literature. Therefore, the aim of the study was to investigate the pharmacokinetics and pharmacodynamics of different intraperitoneal insulin boluses. There is a lack of studies comparing the insulin appearance in the systemic circulation after intraperitoneal compared with subcutaneous insulin delivery. Thus, we also aimed for a comparison with the subcutaneous route.

**Research design and methods:**

Eight anesthetized, non-diabetic pigs were given three different intraperitoneal insulin boluses (2, 5 and 10 U). The order of boluses for the last six pigs was randomized. Endogenous insulin and glucagon release were suppressed by repeated somatostatin analog injections. The first pig was used to identify the infusion rate of glucose to maintain stable glucose values throughout the experiment. The estimated difference between insulin boluses was compared using two-way analysis of variance (GraphPad Prism V.8).

In addition, a trial of three pigs which received subcutaneous insulin boluses was included for comparison with intraperitoneal insulin boluses.

**Results:**

Decreased mean blood glucose levels were observed after 5 and 10 U intraperitoneal insulin boluses compared with the 2 U boluses. No changes in circulating insulin levels were observed after the 2 and 5 U intraperitoneal boluses, while increased circulating insulin levels were observed after the 10 U intraperitoneal boluses. Subcutaneously injected insulin resulted in higher values of circulating insulin compared with the corresponding intraperitoneal boluses.

**Conclusions:**

Smaller intraperitoneal boluses of insulin have an effect on circulating glucose levels without increasing insulin levels in the systemic circulation. By increasing the insulin bolus, a major increase in circulating insulin was observed, with a minor additive effect on circulating glucose levels. This is compatible with a close to 100% first-pass effect in the liver after smaller intraperitoneal boluses. Subcutaneous insulin boluses markedly increased circulating insulin levels.

**Significance of this study**

**What is already known about this subject?**

- After subcutaneous insulin boluses there is a dose-dependent increase in circulating insulin levels. This has until yet not been properly studied after intraperitoneal insulin delivery.

**What are the new findings?**

- The increase in circulating insulin levels as well as the glucose-lowering effect of intraperitoneally delivered insulin appears to be non-linear.
- We observed a close to 100% first-pass effect in the liver after the smaller (2 and 5 U) IP insulin boluses with hardly any insulin appearing in the systemic circulation concomitant with a major effect on circulating glucose levels.
- Larger (10 U) intraperitoneal insulin boluses increased systemic circulating insulin levels with only a minor further decrease in glucose levels.

**How might these results change the focus of research or clinical practice?**

- The present results underline the importance of the liver in glucose homeostasis, that a first-pass effect after a smaller intraperitoneal insulin dose approaches 100%, that good glucose control with normal circulating insulin levels could be possible, and that the glucose-lowering effect of insulin achieved in extrahepatic tissues throughout the body is minor compared with the hepatic effect.
- The present results underline that the algorithms in an artificial pancreas with intraperitoneal insulin delivery need to reflect the non-linear relationship between intraperitoneal insulin delivered and the subsequent effect on systemic circulating glucose levels.

## Introduction

Insulin is the major hormone affecting the circulating blood glucose levels. An autoimmune destruction of the insulin-producing beta cells in the pancreas is the cause of diabetes mellitus type 1 (DM1). Thus, patients with DM1 are totally dependent on an external supply of insulin. Usually, insulin is supplied subcutaneously, either by multiple daily insulin injections or by continuous subcutaneous insulin infusion (CSII) by an insulin pump. From a theoretical point of view, intraperitoneal delivery of insulin mimics the normal physiology more closely than subcutaneous insulin delivery.^1^ Animal studies suggest that insulin administration in the portal vein is comparable to endogenous insulin delivery from the pancreas and mimics the normal physiological portal and systematic insulin levels and effects in the liver.^2^ However, as intraportal insulin delivery in humans probably carries an unacceptable risk of complications, continuous intraperitoneal insulin infusion has been applied instead of CSII in patients with either severe subcutaneous insulin resistance or brittle diabetes, and with some improvement of HbA1c.^3^

Intraperitoneally administrated insulin reaches maximum circulating insulin levels faster and decreases faster than after subcutaneous insulin delivery.^3 4^ Intraperitoneal insulin administration also seems to decrease the risk of hypoglycemia.^5^

The last decade has seen multiple attempts to make an artificial pancreas (AP), that is, a fully automated delivery of insulin in patients with DM1. So far, almost all attempts are based on the double subcutaneous approach, that is, both continuous glucose measurements (CGM) and insulin delivery in subcutaneous tissue. Unfortunately, this approach is hampered by significant delays both in subcutaneous CGM and in particular in the glucose-lowering effect of subcutaneously delivered insulin.^1^ Thus, until yet, only hybrid APs are available on the marked. Currently, patients must inform their hybrid AP about the carbohydrate content of their meals and the device will estimate the bolus of insulin to be given.

Aiming to reduce the delays inherent in a double subcutaneous AP and to be able to make a true AP without the need for multiple daily interventions by the users, our research group works on a double intraperitoneal approach for an AP.^1^

To develop the control algorithms for such an AP with intraperitoneal delivery of insulin we need detailed information on the dynamics of intraperitoneal insulin boluses and the effect on glucose levels in the systemic circulation. With that aim, we performed an animal study with frequently repeated measurements of insulin and circulating glucose levels after intraperitoneal insulin boluses of different sizes.

## Research design and methods

### Animals

#### Main trial

Between January 2017 and August 2018, eight juvenile, non-diabetic, cross-bred pigs (50% Landswine, 50% Yorkshire) approximately 3 months of age (one male (36.2 kg) and seven females (39.5±2.7 kg)) were brought from the same local farmer approximately 1 week before the trials and acclimatized to the staff and new environment. Whenever possible, the pigs were kept in groups in a common stall (11.2 m^2^) with concrete floor covered with woodchips. In every stall, a heating lamp was provided. In the facility, a day-night photoperiod (night: 21:00–05:00, dawn: 05:00–07:00, day: 07:00–19:00, dusk: 19:00–21:00) was maintained at 22°C and a relative humidity of 45%±5%. The pigs were fed standard food (Format Vekst 100, Felleskjøpet, Norway) and fresh water was available *ad libitum*. Food was removed 17 hours before the start of the trial while water was available until anesthesia was initiated.

#### Additional trial

We also performed an additional trial to investigate subcutaneous insulin dynamics and effect on blood glucose levels, performed under similar conditions. For more details, see online supplemental material section 'Additional trial'. In short, between May 2019 and January 2020, three juvenile, non-diabetic, cross-bred male pigs (50% Landswine, 50% Yorkshire) approximately 3 months of age weighing 39.5±4.3 kg were brought from the same local farmer and kept in the conditions as in the main trial.

10.1136/bmjdrc-2020-001929.supp1 Supplementary data

### Intervention and randomization

In the main trial, the first pig was used to obtain information about optimal infusion rate of glucose to maintain stable glucose levels throughout the trial days. The second pig was used to confirm the chosen infusion rate. The remaining six pigs were used to study the pharmacokinetics and pharmacodynamics of intraperitoneally delivered insulin boluses. However, all pigs received boluses of 2, 5 and 10 U of insulin (1 U/s) in the upper right quadrant of the intraperitoneal cavity. The order of insulin boluses for the first two pigs was 2, 5 and 10 U. The order of boluses for the last six pigs was randomized.^6^ There were at least 2 hours and 30 min between each bolus.

In the additional trial, two pigs received 10 U subcutaneous insulin boluses and one pig received a 5 U subcutaneous insulin bolus. All subcutaneous insulin boluses were performed as the first bolus of the day.

### Anesthesia

In both the main trial and the additional trial, anesthesia was maintained by intravenous infusion of midazolam (0.5 mg/kg/hour) (Accord Healthcare, Middlesex, UK) and fentanyl (7.5 µg/kg/hour) (Actavis Group, Hafnarfjordur, Iceland) and by inhalation of isoflurane (0.5%–2%) (Baxter AS, Oslo, Norway). More detailed information regarding the anesthesia protocol is provided in the online supplemental material.

### Surgical procedure

In both trials, an intra-arterial line was placed in the left carotid artery for blood sampling and monitoring of physiological parameters and an intravenous line was placed in the left internal jugular vein for glucose and fluid infusions. Both catheters were inserted through the same surgical opening.

The catheter for intraperitoneal delivery of insulin by an Animas Vibe insulin pump (Animas, West Chester, Pennsylvania, USA) was inserted through 2–3 cm long caudal to the umbilicus incision in the abdominal wall. The tip of the catheter was inserted intraperitoneally in the upper right region but was not fixed in the stationed position. To avoid coagulation, 150 IU of heparin (LEO Pharma, Ballerup, Denmark) was injected into the intraperitoneal space.

At the end of all trial days, and still under full anesthesia, the pigs were euthanized. More detailed information is provided in the online supplemental material (Surgical procedure).

### Endogenous insulin and glucagon secretion

To suppress the endogenous insulin and glucagon secretion, all pigs received the somatostatin analogs octreotide (Sandostatin 200 µg/mL, Novartis Europharm, UK) and pasireotide (Signifor 0.3 mg/mL, Novartis Europharm).

The somatostatin analogs were injected 1 hour before the first insulin bolus of the day. 0.4 mg octreotide was injected intravenously and 0.3 mg pasireotide was injected subcutaneously. The octreotide injections were repeated hourly and the pasireotide injections were repeated every 3 hours during the trial.

### Glucose level

To prevent hypoglycemia, a continuous venous glucose infusion was provided through the left internal jugular vein and was continued for the duration of the experiment. The blood glucose levels were kept in the range of 4.5–5.5 mmol/L before each insulin bolus was given.

In the first pig, we tested different glucose infusion rates to identify a suitable rate. In the remaining seven pigs, a constant glucose infusion rate of 8 g/hour was used throughout the experiments.

In the additional trial, the glucose infusion rate was increased in one of the pigs during two separate periods due to hypoglycemia. Additionally, all pigs received an intraperitoneal glucagon bolus (150 µg) 40 min after the insulin bolus.

Accordingly, only the circulating insulin levels from the additional trial were compared with the mean circulating insulin levels from the main trial, that is, the blood glucose values from the additional trial were not compared directly to the results from the main trial due to increased glucose infusion rate.

### Insulin boluses

At the start of every trial day, a fresh insulin analog (100 U/mL, NovoRapid, Novo Nordisk, Denmark) was inserted in an insulin pump (Animas Vibe).

In the main trial, all pigs (n=7) received three insulin boluses (2, 5 and 10 U) in the upper right intraperitoneal space. In the additional trial, insulin boluses were injected into the subcutaneous tissues in the left side of the neck. Two pigs received 10 U boluses and one pig received a 5 U bolus.

### Glucose and insulin measurements

For both trials, arterial blood samples for glucose analysis were collected in heparinized syringes (LEO Pharma). Samples were analyzed on a Radiometer ABL 725 blood gas analyzer (Radiometer Medical, Brønshøj, Denmark). All blood samples were placed on ice immediately after extraction from the pigs. Most samples were analyzed consecutively, but some samples were stored on ice for a maximum of 20 min before analysis.

In the main trial, samples were collected 10, 5 and 1 min prior to the first somatostatin analog injection, every 10 min for the first hour after Sandostatin injection, every minute for the first 10 min after the insulin bolus, and every fifth minute thereafter for the next 110 min.

In the additional trial, samples for blood glucose measurements were collected 10, 5 and 1 min prior to first somatostatin analog injection and prior to starting the glucose infusion. Subsequent blood glucose samples were collected 2 min after insulin boluses and thereafter every 5 min for the next 118 min.

In both trials, blood samples for insulin analysis were stored on ice for at least 10 min before centrifugation (10 min at 2.000× rpm in a refrigerated centrifuge). Plasma was collected from the samples immediately after centrifugation and transferred into empty Eppendorf Tubes and temporarily stored at −20°C. At the end of each trial day, plasma samples were stored at −80°C.

Plasma insulin was analyzed as singles by Iso-Insulin ELISA kit (10-1128-01, Mercodia, Uppsala, Sweden). Suppression of endogenous insulin secretion was verified by analyses of Porcine Insulin ELISA (10-1200-01, Mercodia) according to the manufacturer’s protocol. The results were converted from mU/L to pmol/L by a conversion factor 6, as recommended by the manufacturer. The lowest detectable insulin concentration for the Iso-Insulin ELISA kit was <3.0 mU/L (18 pmol/L) and for Porcine Insulin ELISA was <2.3 mU/L (13.8 pmol/L).

### Statistical analysis

From the main trial, data from the last seven pigs were used for statistical analysis. We assumed the glucose levels at −5 min to be the baseline glucose levels at the start before each bolus (online supplemental figure S1).

Delta values collected from the main trial with different insulin boluses (2, 5 and 10 U) in the pigs were analyzed. In order to estimate possible significant differences in circulating insulin and blood glucose levels after IP insulin boluses, a two-way repeated measures analysis of variance was performed. Treatment and time were the sources of variation. Tukey’s multiple comparisons test was used for distinguishing comparisons between different insulin boluses. All non-measurable insulin values were set at 18 pmol/L. All treatments are compared as models; therefore, comparison between unequal groups is allowed. Statistics were performed with GraphPad Prism V.8 Statistics. All values are given as mean±SD, unless stated otherwise. Differences between the groups were considered significant if p≤0.05.

## Results

### Pilot experiment

The first pig was used for a pilot experiment as we had no information about the necessary glucose infusion rate to achieve acceptable glucose values throughout the trial days. Based on the results from the first pig, a continuous glucose infusion of 8 g/hour (200 mg/mL at 40 mL/hour) was used throughout the rest of the trial.

Octreotide and pasireotide had the expected effect on insulin levels, as there were no detectable endogenous insulin levels (<13.8 pmol/L) during the experiments. Therefore, the same protocol was used for all experiments.

### Glucose level

In the main trial, the mean blood glucose level at the start of the 2, 5 and 10 U insulin boluses was 5.07±0.11, 5.38±0.06 and 5.31±0.06 mmol/L, respectively (online supplemental figure S1A).

**Figure 1:**
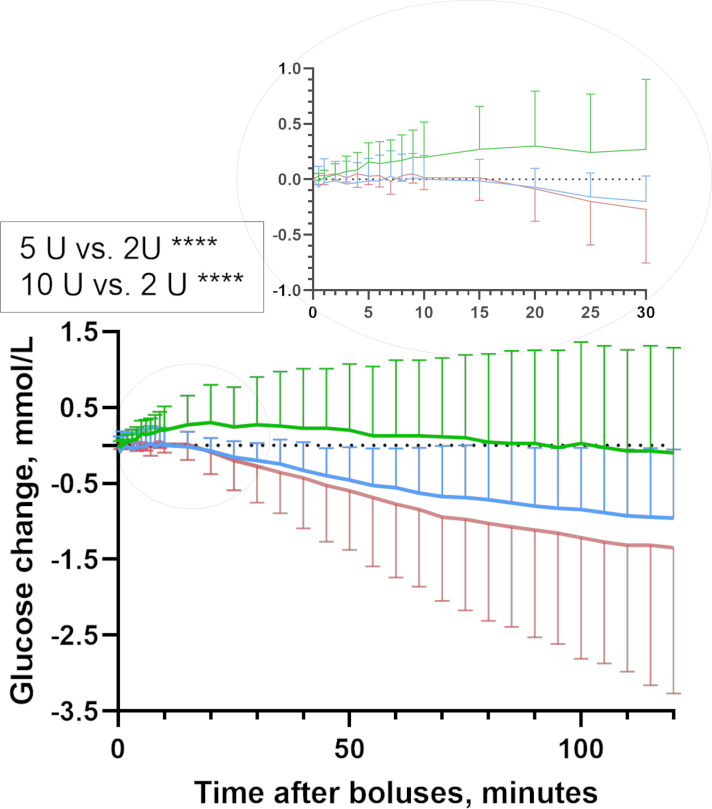
Glucose dynamics. Estimated blood glucose change for the 120 min after intraperitoneal insulin delivery in pigs (mean, SD). The 5 and 10 U intraperitoneal bolus gave significantly higher glucose elevations compared with 2 U intraperitoneal bolus. Insulin boluses: 2 U intraperitoneal insulin bolus (n=7, green line), 5 U intraperitoneal insulin bolus (n=7, blue line) and 10 U intraperitoneal insulin bolus (n=7, red line).

The estimated mean blood glucose level after the 5 and 10 U intraperitoneal insulin boluses was significantly decreased (mean±SE) by 0.48±0.07 and 0.61±0.07 mmol/L (95% CI 0.31 to 0.65 and 95% CI 0.44 to 0.79, respectively, p<0.0001) compared with the mean blood glucose level after the 2 U insulin boluses (figure 1). However, the mean blood glucose level after the 10 U intraperitoneal insulin boluses changed only by 0.13±0.07 mmol/L (95% CI −0.04 to 0.30, p=0.184) and was not different from the mean blood glucose level after the 5 U intraperitoneal insulin boluses.

### Insulin level

**Figure 2:**
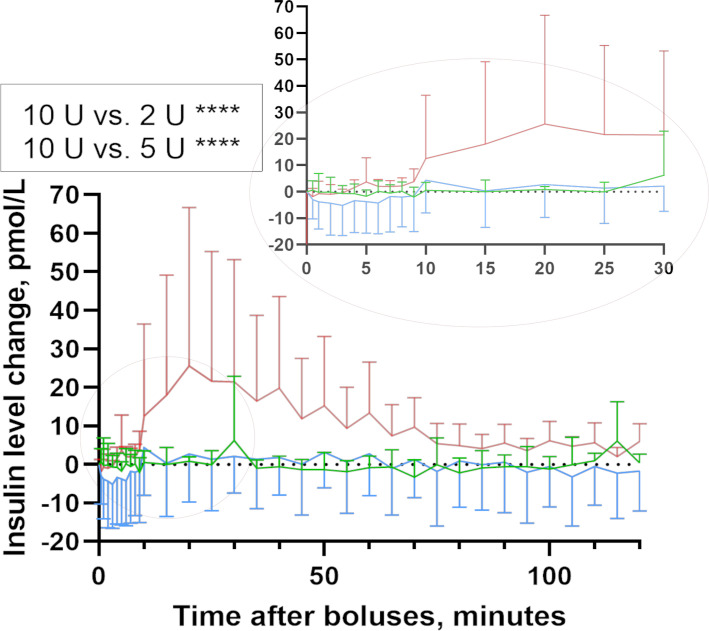
Insulin dynamics. Estimated insulin change for the 120 min after intraperitoneal insulin delivery in pigs (mean, SD). The 10 U intraperitoneal bolus gave significantly higher insulin elevation compared with 2 and 5 U intraperitoneal boluses. Insulin boluses: 2 U intraperitoneal insulin bolus (n=7, green line), 5 U intraperitoneal insulin bolus (n=7, blue line) and 10 U intraperitoneal insulin bolus (n=7, red line).

No difference in circulating mean insulin level was observed after the 2 and 5 U intraperitoneal insulin boluses while after the 10 U intraperitoneal insulin boluses, the mean insulin level started to increase after 10 min. The mean insulin level after the 10 U intraperitoneal insulin boluses was significantly increased by (mean±SE) 7.89±1.07 and 8.37±1.07 pmol/L compared with the mean insulin level after the 2 and 5 U intraperitoneal insulin boluses (95% CI 1.99 to 5.37 and 95% CI 5.86 to 10.89, respectively, p<0.0001) (figure 2). Mean insulin levels after 5 U compared with 2 U intraperitoneal insulin boluses were not different (p=0.89).

In the additional trial, the insulin level was increased 5 min after 5 U subcutaneous insulin bolus (n=1) and the mean insulin level was increased 5 min after 10 U subcutaneous insulin boluses (n=2) (online supplemental figure S4).

In the main trial, all insulin samples were run in singles with a coefficient of variation (CV) <5%. Interassay CV for Porcine Insulin was 4.1%, 4.3% and 3.3% for 5.04, 17.6 and 55.4 mU/L standards, respectively. Interassay CV for Iso-Insulin was 4.9% and 4.7% for 9.84 and 60.7 mU/L standards, respectively.

### Comparison of intraperitoneal and subcutaneous insulin boluses

**Figure 3:**
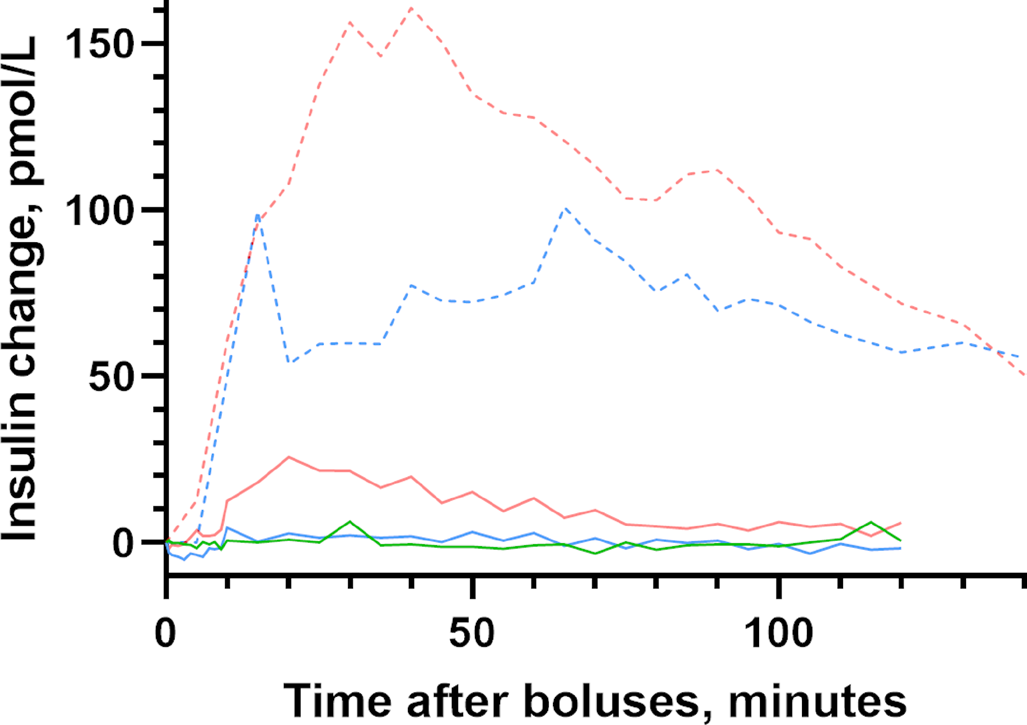
Insulin change after intraperitoneal and subcutaneous insulin boluses. Estimated insulin changes for the 120 min after intraperitoneal insulin delivery and for the 140 min after subcutaneous insulin delivery in pigs. Statistical testing was not provided based on limited number of included animals. Insulin boluses: 2 U intraperitoneal insulin bolus (n=7, green line), 5 U intraperitoneal insulin bolus (n=7, blue line), 10 U intraperitoneal insulin bolus (n=7, red line), 5 U subcutaneous insulin bolus (n=1, blue dashed line) and 10 U subcutaneous insulin bolus (n=2, red dashed line).

The mean circulating insulin level increased more after subcutaneous insulin boluses compared with intraperitoneal insulin boluses (p value not calculated due to low numbers) (online supplemental figures S2A, B, S5 and S6). Further, after the 10 U intraperitoneal insulin boluses, the mean circulating insulin level was close to baseline after 80 min, whereas after the 5 and 10 U subcutaneous insulin boluses, the insulin levels were still elevated after 140 min (figure 3).

## Discussion

The results of this animal trial indicate that: (1) after intraperitoneal insulin delivery there is a threshold dose before insulin appears in the systemic circulation, and (2) there is clear non-linear relationship between the intraperitoneal insulin dose and its glucose-decreasing effect. This is in contrast to the effect of subcutaneous insulin boluses seen in humans, where a linear association between increasing insulin doses and decreasing blood glucose is observed.^7^

In the additional trial, higher circulating insulin levels were observed after the subcutaneous compared with the intraperitoneal insulin boluses. Markedly increased circulating insulin levels after subcutaneous delivery had no effect or a much smaller effect on circulating insulin levels after intraperitoneal delivery of the same insulin bolus.

Previous human studies on intraperitoneal insulin infusion in humans did not report any major difference between circulating insulin levels when intraperitoneal was compared with either subcutaneous insulin boluses (0.2 U/kg)^8^ or insulin infusion (5 U for 30 min followed by 2 U/hour for 3 hours).^9^ One possible explanation may be that our data are generated from anesthetized pigs while the previous data are from human studies. Our data are also limited by the small number of pigs and insulin boluses used and need to be confirmed in additional studies with preferably a larger number of animals. We observed in our pig trials that both 2 and 5 U intraperitoneal insulin boluses did not increase the mean circulating insulin level, while the 10 U intraperitoneal insulin bolus significantly increased the mean circulating insulin level. This indicates that more or less all intraperitoneally delivered insulin, at least after the two smaller boluses, is absorbed into the portal circulation and that there is a substantial hepatic first-pass effect absorbing more or less all insulin delivered to the liver from the portal vein.^10^

A hepatic first-pass effect of up to 80% has been shown in an in vitro model.^11^ Our data are consistent with that observation. Actually, our data indicate a close to 100% first-pass effect of insulin in the liver before the mechanism for hepatic insulin absorption is saturated. The liver is the major organ involved in glucose homeostasis. Our observation that there is hardly any difference in the glucose-lowering effect after the 5 and 10 U intraperitoneal insulin boluses is probably explained by this first-pass effect in the liver when the insulin delivery from the portal vein reaches a certain level. When this level of insulin is reached, the liver cannot absorb more glucose per unit time, that is, the hepatic capacity for glucose disposal is saturated and further increase in total body glucose disposal can only be achieved in extrahepatic tissues such as muscle and fat. This is illustrated by the fact that despite doubling the intraperitoneal insulin boluses from 5 to 10 U hardly any further decrease in circulating glucose is observed. This is compatible with the liver being saturated with insulin after a 5 U intraperitoneal bolus and no further effect on hepatic glucose disposal is achievable despite increasing intraperitoneal insulin doses. Similar results were observed in human studies where higher insulin doses were provided during the intraperitoneal insulin treatment without any increase in hypoglycemic events, as compared with the subcutaneous insulin delivery.^12^

Increasing the intraperitoneal insulin boluses to 10 U means that circulating insulin levels increase while the glucose-lowering effect is minimally increased. This illustrates that what can be achieved by insulin-mediated glucose disposal by other tissues such as fat and muscle is quite limited compared with the hepatic effect.^13–15^

We cannot exclude that the order of the boluses may influence the results. First, we were not able to look into this due to the low number of animals in our study, but we observed that during the trial day (8 hours), the amount of intraperitoneal fluid increased. Therefore, we hypothesize that increased amount of intraperitoneal fluid may reduce the speed of insulin absorption as previously suggested.^16^ Second, as we gave the largest bolus (10 U) first, we trespassed the saturation in the liver, and the excess was distributed peripherally, and there may then be a larger chance that the next boluses of 2 and 5 U will be distributed peripherally as well, because of a possible lasting effect of the 10 U bolus on the hepatic insulin saturation. If so, the opposite may be the issue, when we give 2 U as the first bolus of the day. In that case, theoretically a smaller proportion of the subsequent bolus of 5 or 10 U will be distributed peripherally, because most of that insulin bolus will be bound in the liver, because the previous 2 U insulin bolus far from saturated the liver. However, we do not know how long insulin exerts its effect in the liver and the hepatic saturation is repealed.

The fact that the more or less linear relationship between insulin dose and effect on blood glucose levels observed after subcutaneous insulin delivery is quite different when insulin is delivered intraperitoneally, illustrates that the steering algorithms for an AP with subcutaneous insulin delivery cannot be transferred directly to an AP with intraperitoneal insulin delivery. This means that the mathematical model used in the controller (or the simulator we would test the controller on) should probably include information about this strong non-linearity. It is noteworthy that, when given intraperitoneally, a substantial effect on blood glucose levels can be achieved without any increase in circulating insulin levels and that a doubling of a medium insulin dose hardly induces further glucose-lowering effect.

### Strengths and limitations of the study

The strengths of our trial are (1) frequent blood sampling before and for a relatively long period after each of the insulin boluses, (2) randomized order of boluses (in 6 out of 7 pigs), (3) equal age and gender of the pigs, (4) verified complete suppression of endogenous insulin secretion, (5) equal glucose levels at the initiation of the experiments and equal glucose infusion rate which makes the observed glucose effects more trustworthy (main trial), and (6) data from intraperitoneal insulin boluses were compared with subcutaneous insulin boluses with similar sampling times and similar protocol through the trials.

Among the limitations are (1) weights of the animals varied somewhat while insulin boluses were fixed, (2) limited number of included animals, especially in the additional trial with subcutaneous insulin boluses, (3) animals were anesthetized during the study, therefore obtained data do not reflect awakening animal dynamics of insulin absorption and effects, and (4) during the experiments animals accumulated different amounts of intraperitoneal fluid possibly affecting insulin absorption and effect.

## Conclusions

In pigs, small to medium intraperitoneal insulin boluses (2 and 5 U) decrease circulating glucose levels without increasing insulin levels in the systemic circulation. By increasing the insulin bolus further (to 10 U), we observed a major increase in circulating insulin levels while only a minor additional lowering of blood glucose levels was observed. This is compatible with a close to complete first-pass effect in the liver after small to medium-sized intraperitoneal insulin boluses.
