# Supplementary material for: Intraperitoneal insulin administration in pigs: effect on circulating insulin and glucose levels
Source: BMJ Open Diabetes Res Care. 2021 Jan 15;9(1):e001929. doi: 10.1136/bmjdrc-2020-001929 (PMC7813410; doi:10.1136/bmjdrc-2020-001929)
Supplement: Supplementary data [file bmjdrc-2020-001929supp001.pdf]

Online supplementary materials

# Title ”Intraperitoneal insulin administration in pigs: Effect on circulating insulin and glucose levels”

## 1 RESEARCH DESIGN AND METHODS

### 1.1 Anaesthesia

The pigs were premedicated with an intramuscular injection of 4 mg diazepam (Stesolid®, Actavis Group, Hafnarfjordur, Iceland), 160 mg azaperone (Stresnil®, Eli Lilly Regional Operations GmbH, Austria) and 750 mg ketamine (Ketalar®, Pfizer AS, Norway), while in a stall. The pigs were carried to the operation room and weighed. An aurical vein was cannulated and anaesthesia was induced with intravenous injections of 1 mg atropine (Takeda AS, Asker, Norway), 150 – 250 µg fentanyl (Actavis Group, Hafnarfjordur, Iceland), 75 – 125 mg thiopental (VUAB Pharma AS, Roztoky, Czech Republic) and 150 – 250 mg ketalar (Ketalar®, Pfizer AS, Norway). The same method was used in our previous animal trials [1].

The pigs were intubated in the lateral position and mechanically ventilated and monitored on an anaesthesia machine (Aisys, GE Healthcare Technologies, Oslo). Anaesthesia was maintained by intravenous infusion of midazolam (0.5 mg/kg/h) (Accord Healthcare Limited, Middlesex, UK) and fentanyl (7.5 µg/kg/h) (Actavis Group, Hafnarfjordur, Iceland) and by inhalation of isoflurane (0.5 – 2 %) (Baxter AS, Oslo, Norway). Room temperature was around 20 degrees Celsius. The body temperature of the pigs was monitored, and a heating blanket was used when necessary.

The pigs received two IV infusions of antibiotic (Cefalotin, Villerton Invest SA, Luxembourg) during the experiments; 2 g immediately after the pigs were anaesthetised and 1 g after 4 hours. Heparin (150 IE) (LEO Pharma A/S, Ballerup, Denmark) were injected in the peritoneal space through the opening for catheter insertion. Fluid balance was achieved by continuous IV infusion of Ringer’s acetate with individual adjustments to achieve stable blood pressure. The pigs also received IV fluid through antibiotics, glucose infusion and when the catheters were flushed after every blood sample.

## 1.2 Additional trial.

The additional trials were performed for a non-related study.

In contrast to the main study, two of the pigs in the additional study received an increased glucose infusion rate 20 and 70 minutes after the SC insulin bolus, respectively. All three pigs were also given an intraperitoneal (IP) glucagon bolus 40 minutes after the SC insulin injection.

The total fluid loss during the experiments is not known, but estimates suggest that the pigs were in positive fluid balance, even at the lower infusion regimen.

## 1.3 Surgical procedure

The pig was scrubbed with chlorhexidine (20 mg/ml) (Sage Products, The Netherlands) and covered with an operation blanket. An intra-arterial line was placed in the left carotid artery for blood sampling and monitoring of physiological parameters and an IV line was placed in the left internal jugular vein for glucose and fluid infusions. Both catheters were inserted through the same cut-down.

Catheter for IP insulin infusion by an insulin pump was inserted through a 2 – 3 cm long caudal-umbilicus incision in the abdominal wall. The tip of the catheter was inserted in the upper right IP region but was not fixed in the stationed position. To avoid coagulation heparin (150 IE) (LEO Pharma A/S, Ballerup, Denmark) was injected in IP space. Opening was closed with medical clamps and catheter was fixed with tape. The bladder was exposed through a small, low laparotomy for the insertion of a bladder catheter. Both cuts were made with a thermocauter to minimise bleeding into the abdominal cavity.

At the end of the experiments, and under full anaesthesia, the pigs were euthanised with an IV overdose of pentobarbital (minimum 100 mg/kg) (pentobarbital NAF, Apotek, Lørenskog, Norway).

# 2 RESULTS

## 2.1 Main trial

### 2.1.1 Raw blood glucose levels after IP insulin boluses

Raw blood glucose levels were measured for 120 minutes after administration of insulin boluses in pigs (n = 7). Fig S1a presents data from all time points, whereas Fig S1b shows specifically data from first 30 minutes.

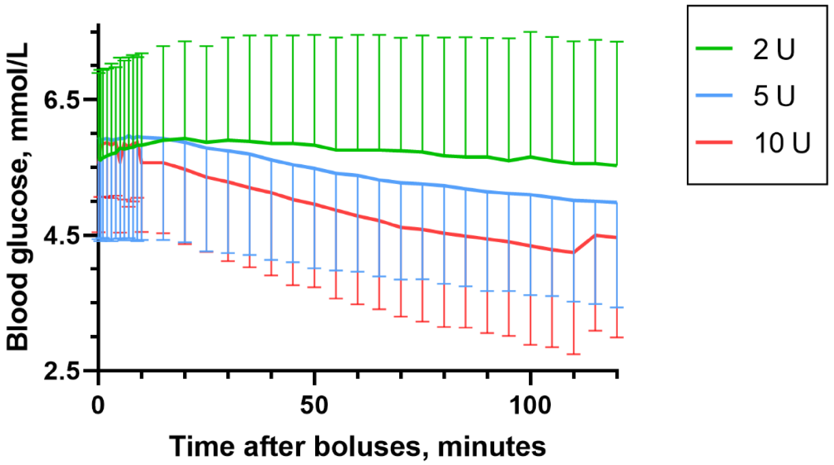


**Figure S1a.** Blood glucose levels after IP insulin boluses.

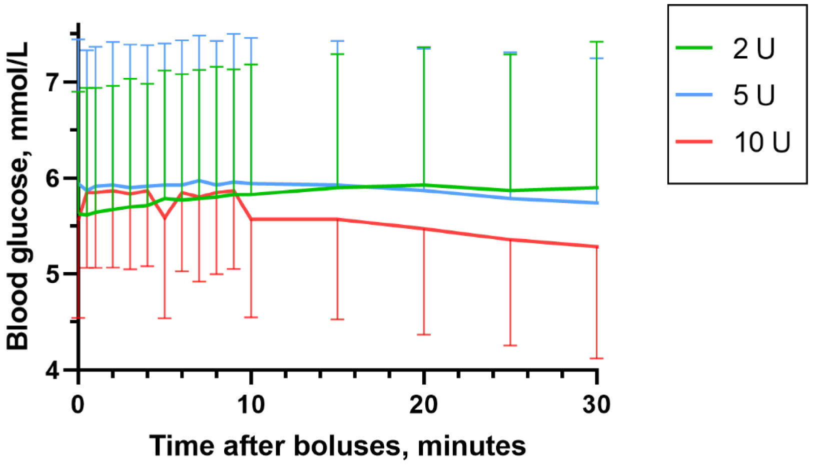


**Figure S1b.** Blood glucose levels for the first 30 minutes after IP insulin boluses.

### 2.1.2 Raw insulin levels after IP insulin boluses

Raw insulin levels were measured for 120 minutes after administration of insulin boluses in pigs (n = 7).

Fig S2a presents data from all time points, whereas Fig S2b shows specifically data from first 30 minutes.

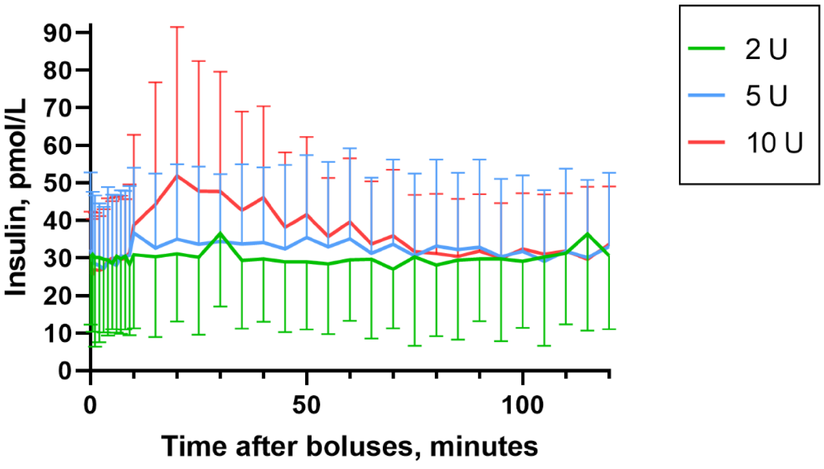


**Figure S2a.** Insulin levels after IP insulin boluses.

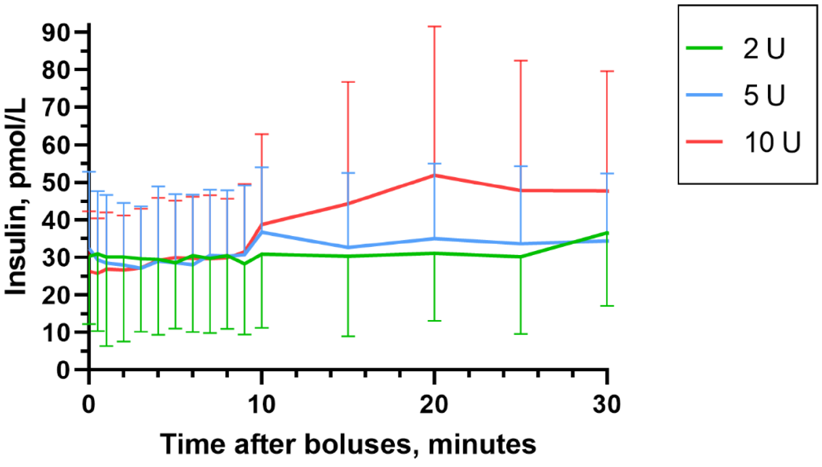


**Figure S2b.** Insulin levels for the first 30 minutes after IP insulin boluses.

## 2.2 Additional trial

### 2.2.1 Raw blood glucose levels after SC insulin boluses

Data from raw blood glucose levels are presented in Fig S3. The glucose infusion rate was increased in one of the pigs during two separate periods due to hypoglycaemia. Additionally, all pigs (n = 3) received an IP glucagon bolus (150 µg) 40 minutes after the insulin bolus.

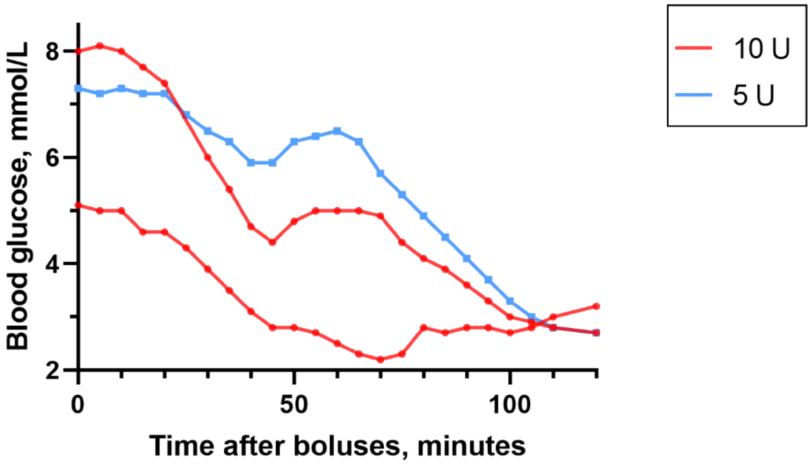


**Figure S3.** Blood glucose levels after SC insulin boluses.

### 2.2.2 Raw insulin levels after SC insulin boluses

Data from raw insulin levels are presented in Fig S4.

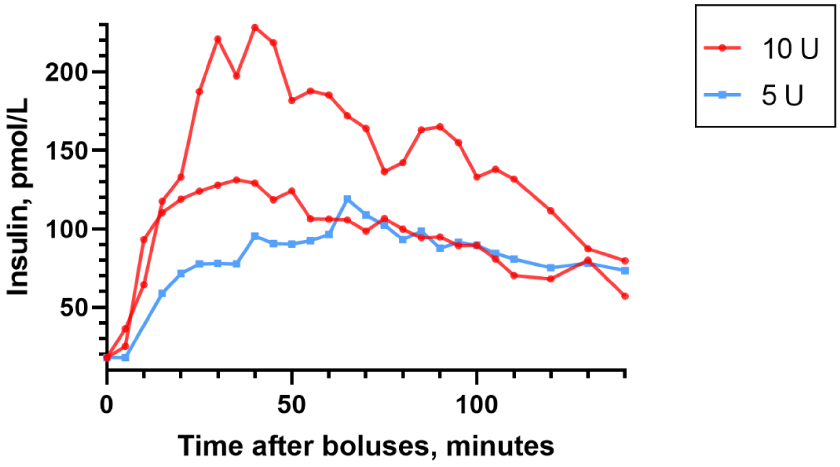


**Figure S4.** Insulin levels after SC insulin boluses.

Duration of presented measurements differ between blood glucose and insulin levels due the start of glucose infusion in the pigs 120 minutes after insulin boluses.

### 2.2.3 Insulin delta values after SC insulin boluses

Insulin delta values are presented in Fig S5.

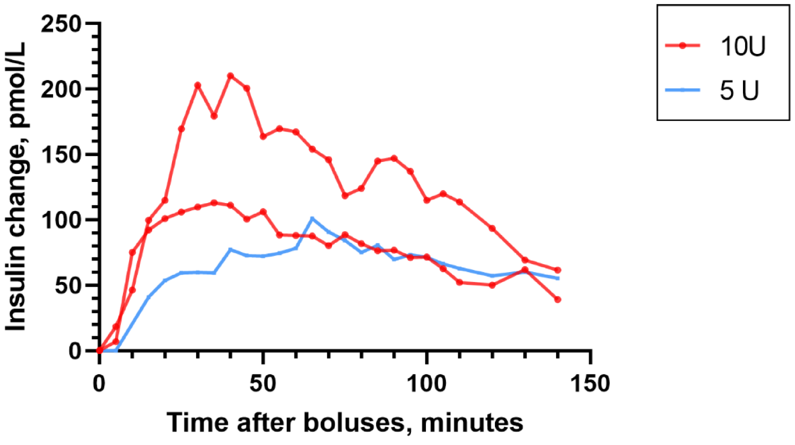


**Figure S5.** Insulin levels change after SC insulin boluses.

## 2.3 Comparison between Intraperitoneal and subcutaneous insulin boluses

### 2.3.1 Raw data comparison between IP and SC insulin boluses

Raw data comparison between IP insulin boluses (n = 7) and SC insulin boluses is presented in Fig S6.

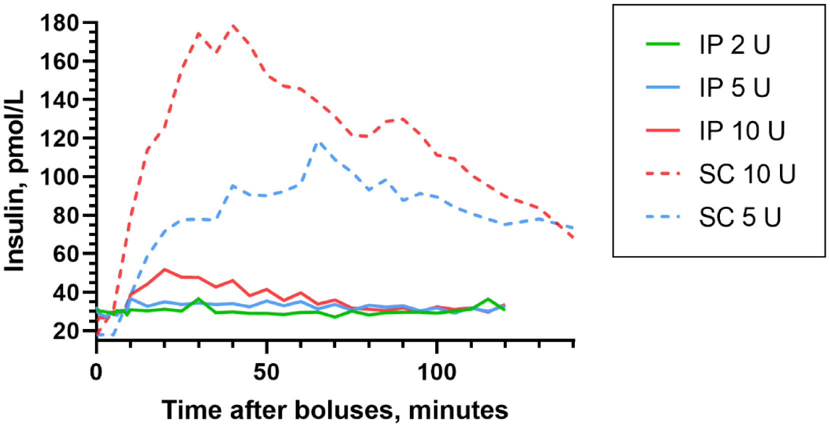


**Figure S6.** Insulin levels after IP and SC insulin boluses.

**REFERENCES**

1. Åm, M.K., et al., *Intraperitoneal and subcutaneous glucagon delivery in anaesthetized pigs: effects on circulating glucagon and glucose levels.* Sci Rep, 2020. **10**(1): p. 13735.
